# Japan Society of Clinical Oncology Clinical Practice Guidelines 2017 for fertility preservation in childhood, adolescent, and young adult cancer patients: part 2

**DOI:** 10.1007/s10147-021-02076-7

**Published:** 2022-01-13

**Authors:** Akiko Tozawa, Fuminori Kimura, Yasushi Takai, Takeshi Nakajima, Kimio Ushijima, Hiroaki Kobayashi, Toyomi Satoh, Miyuki Harada, Kohei Sugimoto, Shigehira Saji, Chikako Shimizu, Kyoko Akiyama, Hiroko Bando, Akira Kuwahara, Tatsuro Furui, Hiroshi Okada, Koji Kawai, Nobuo Shinohara, Koichi Nagao, Michio Kitajima, Souichi Suenobu, Toshinori Soejima, Mitsuru Miyachi, Yoko Miyoshi, Akihiro Yoneda, Akihito Horie, Yasushi Ishida, Noriko Usui, Yoshinobu Kanda, Nobuharu Fujii, Makoto Endo, Robert Nakayama, Manabu Hoshi, Tsukasa Yonemoto, Chikako Kiyotani, Natsuko Okita, Eishi Baba, Manabu Muto, Iwaho Kikuchi, Ken-ichirou Morishige, Koichiro Tsugawa, Hiroyuki Nishiyama, Hajime Hosoi, Mitsune Tanimoto, Akira Kawai, Kazuhiko Sugiyama, Narikazu Boku, Masato Yonemura, Naoko Hayashi, Daisuke Aoki, Nao Suzuki, Yutaka Osuga

**Affiliations:** 1 Department of Obstetrics and Gynecology, St. Marianna University School of Medicine, 2-16-1 Sugao, Miyamae-ku, Kawasaki-shi, Kawasaki, Kanagawa 216-8511 Japan; 2 Department of Obstetrics and Gynecology, Shiga University of Medical Science, Seta Tsukinowa-Cho Otsu, Shiga, 520-2192 Japan; 3 Department of Obstetrics and Gynecology Saitama Medical Center, Saitama Medical University, 1981 Kamoda, Kawagoe City, Saitama 350-3550 Japan; 4 Department of Endoscopy, Gastrointestinal Endoscopy Division, National Cancer Center Hospital, 5-1-1 Tsukiji, Chuo-ku, Tokyo, 104-0045 Japan; 5 Department of Obstetrics and Gynecology, Kurume University School of Medicine, 67 Asahi-machi, Kurume, Fukuoka, 830-0011 Japan; 6 Department of Obstetrics and Gynecology, Graduate School of Medical and Dental Sciences, Kagoshima University, 8-35-1 Sakuragaoka, Kagoshima, 890-8520 Japan; 7 Department of Obstetrics and Gynecology, Faculty of Medicine, University of Tsukuba, Tennoudai, Tsukuba, Ibaraki 305-8575 Japan; 8 Department of Obstetrics and Gynecology, Graduate School of Medicine, The University of Tokyo, 7-3-1, Hongo, Bunkyo, Tokyo, 113-8655 Japan; 9 International Center for Reproductive Medicine, Dokkyo Medical University Saitama Medical Center, 2-1-50 Minamikoshigaya, Koshigaya, Saitama 343-8555 Japan; 10 Department of Medical Oncology, Fukushima Medical University, 1 Hikarigaoka, Fukushima City, Fukushima 960-1295 Japan; 11 Department of Breast and Medical Oncology, National Center for Global Health and Medicine, 1-21-1 Toyama, Shinjuku-ku, Tokyo, 162-8655 Japan; 12 Department of Breast and Endocrine Surgery, St. Marianna University School of Medicine, 2-16-1 Sugao, Miyamae, Kawasaki, Kanagawa 216-8511 Japan; 13 Department of Breast and Endocrine Surgery, Faculty of Medicine, University of Tsukuba, Tennoudai, Tsukuba, Ibaraki 305-8575 Japan; 14 Department of Obstetrics and Gynecology, Graduate School of Biomedical Sciences, Tokushima University, 3-18-15, Kuramoto-cho, Tokushima, 770-8503 Japan; 15 Department of Obstetrics and Gynecology, Gifu University Graduate School of Medicine, 1-1, Yanagido, Gifu City, Gifu 501-1194 Japan; 16 Department of Urology, International University of Health and Welfare, 852, Hatakeda Narita, Chiba, 286-0124 Japan; 17 Department of Renal and Genitourinary Surgery, Hokkaido University Graduate School of Medicine, Kita 15Nishi 7, Kita-ku, Sapporo, Hokkaido 060-8638 Japan; 18 Department of Urology, Toho University Faculty of Medicine, 6-11-1, Omori-Nishi, Ota-ku, Tokyo, 143-8541 Japan; 19 Department of Obstetrics and Gynecology, Nagasaki University Graduate School of Biomedical Sciences, Nagasaki, Japan; 20 Division of General Pediatrics and Emergency Medicine, Department of Pediatrics, Oita University Faculty of Medicine, 1-1 Idaigaoka, Hasama, Yufu, Oita, 879-5593 Japan; 21 Department of Radiation Oncology, Kobe Proton Center, 1-6-8, Minatojima-minamimachi, Chuo-ku, Kobe City, Hyogo 650-0047 Japan; 22 Department of Pediatrics, Kyoto Prefectural University of Medicine Graduate School of Medical Science, 465 Kajii-cho, Hirokoji, Kamigyo-ku, Kyoto, 602-8566 Japan; 23 Department of Health and Nutrition, Faculty of Health and Nutrition, Osaka Shoin Women’s University, 4-2-26 Hishiya-nishi, Higashi-Osaka, Osaka, 577-8550 Japan; 24 Division of Surgery/Surgical Oncology, National Center for Child Health and Development, Tokyo, Japan; 25 Division of Pediatric Surgical Oncology, National Cancer Center Hospital, 5-1-1 Tsukiji, Chuo-ku, Tokyo, 104-0045 Japan; 26 Department of Gynecology and Obstetrics, Kyoto University Graduate School of Medicine, 54 Kawahara-cho, Shogoin, Sakyoku, Kyoto, 606-8507 Japan; 27 Pediatric Medical Center, Ehime Prefectural Central Hospital, 83 Kasuga-machi, Matsuyama City, Ehime 790-0024 Japan; 28 Division of Clinical Oncology and Hematology, Department of Internal Medicine, The Jikei University School of Medicine, 3-19-18 Nishi-Shinbashi, Minato-ku, Tokyo, 105-8461 Japan; 29 Division of Hematology, Department of Medicine, Jichi Medical University, 1-847 Amanuma, Omiya-ku, Saitama City, Saitama 330-8503 Japan; 30 Division of Transfusion, Okayama University Hospital, 2-5-1 Shikata-cho, Kita-ku, Okayama, 700-8558 Japan; 31 Department of Orthopaedic Surgery, Kyushu University, Maidashi 3-1-1, Higashi-ku, Fukuoka, 812-8582 Japan; 32 Department of Orthopaedic Surgery, Keio University School of Medicine, 35 Shinanomachi, Shinjuku, Tokyo, 160-8582 Japan; 33 Department of Orthopedic Surgery, Osaka City University Graduate School of Medicine, 1-4-3 Asahi-Machi, Abeno-Ku, Osaka, 545-8585 Japan; 34 Division of Orthopedic Surgery, Chiba Cancer Center, 666-2 Nitona-cho, Chuo-ku, Chiba, 260-8717 Japan; 35 Children’s Cancer Center, National Center for Child Health and Development, 2-10-1 Okura, Setagaya-ku, Tokyo, 157-8535 Japan; 36 Department of Gastrointestinal Medical Oncology, National Cancer Center Hospital, 5-1-1 Tsukiji, Chuo-ku, Tokyo, 104-0045 Japan; 37 Department of Oncology and Social Medicine, Graduate School of Medical Sciences, Kyushu University, 3-1-1 Maidashi, Higashi-ku, Fukuoka, 812-8582 Japan; 38 Department of Therapeutic Oncology, Kyoto University Graduate School of Medicine, 54 Kawahara-cho, Shogoin, Sakyoku, Kyoto, 606-8507 Japan; 39 Department of Obstetrics and Gynecology, Medical Park Yokohama, 1-1-8, Sakuragi-cho, Yokohama, Kanagawa 231-0062 Japan; 40 Department of Urology, Faculty of Medicine, University of Tsukuba, Tennoudai, Tsukuba, Ibaraki 305-8575 Japan; 41 Department of Nursing, Doshisha Women’s College of Liberal Arts, Kodo, Kyotanabe City, Kyoto 610-0395 Japan; 42 Chugoku Central Hospital, 148-13, Kamiiwanari, Miyuki-cho, Fukuyama City, Hiroshima 720-0001 Japan; 43 Department of Musculoskeletal Oncology and Rehabilitation Medicine, National Cancer Center Hospital, 5-1-1 Tsukiji, Chuo-ku, Tokyo, 104-0045 Japan; 44 Department of Clinical Oncology and Neuro-Oncology Program, Hiroshima University Hospital, 1-2-3 Kasumi, Minami-ku, Hiroshima, 734-8551 Japan; 45 Department of Medical Oncology and General Medicine, IMSUT Hospital, Institute of Medical Science, University of Tokyo, 4-6-1, Shirokanedai, Minato-ku, Tokyo, 108-8639 Japan; 46 Department of Pharmacy, National Cancer Center Hospital East, 6-5-1 Kashiwanoha, Kashiwa-shi, Chiba, Japan; 47 Graduate School of Nursing Science, St Luke’s International University, 10-1 Akashi-cho, Chuo-ku, Tokyo, 104-0044 Japan; 48 Department of Obstetrics and Gynecology, Keio University School of Medicine, 35 Shinanomachi, Shinjuku, Tokyo, 160-8582 Japan

**Keywords:** Practice guideline, Fertility preservation, Childhood, adolescent and young adult (CAYA), Cancer

## Abstract

The Japan Society of Clinical Oncology (JSCO) published the “JSCO Clinical Practice Guidelines 2017 for Fertility Preservation in Childhood, Adolescent, and Young Adult Cancer Patients” in 2017. This was the first guideline in cancer reproductive medicine in Japan. In the field of cancer reproductive medicine, close cooperation between an oncologist and a physician for reproductive medicine is important from before treatment initiation until long after treatment. The guideline takes into consideration disease specificity and provides opinions from the perspective of oncologists and specialists in reproductive medicine that are in line with the current state of the Japanese medical system. It is intended to serve as a reference for medical staff in both fields regarding the availability of fertility preservation therapy before the start of cancer treatment. Appropriate use of this guideline makes it easier to determine whether fertility preservation therapy is feasible and, ultimately, to improve survivorship in childhood, adolescent, and young adult cancer patients. In this article (Part 2), we describe details by organ/system and also for pediatric cancer.

**Supplementary Information:**

The online version contains supplementary material available at 10.1007/s10147-021-02076-7.

## Introduction

Here, we present Part 2 of the Japanese Society of Clinical Oncology (JSCO) Clinical Practice Guidelines 2017 for Fertility Preservation in Childhood, Adolescent, and Young Adult (CAYA) Cancer Patients. This part of the guidelines addresses important clinical questions and provides answers for fertility preservation in CAYA cancer patients according to the organ/system or age group affected by the cancer. The objectives are almost the same as those of Part 1 of the guideline [1], and almost the same methods were used to develop the recommendations.

## Development and grading of guideline recommendations

Drafts written by individual members of the Guideline Working Committee were reviewed by the field subcommittee and then cross-reviewed by other field subcommittees. The guideline recommendations and the recommendation grades were finalized by unanimous consent of core members, who consisted of the chair, sub-chair, supervisors (one oncologist and one reproductive specialist), and one representative member each from nursing, pharmaceutical, and other fields. The definitions of the recommendation grades used in these guidelines are provided in Table 1. A Working Group was established in November 2015. After a consensus meeting in October 2016, external opinions (from 23 academic societies and 6 patient associations) were summarized. After an external assessment based on Appraisal of Guidelines for Research & Evaluation (AGREE) II by the Cancer Therapy Guideline Appraisal Committee, the final manuscript was prepared in June 2017. The Working Group comprised specialists in the 8 target areas covered by this guideline—female reproductive system, mammary glands, urinary tract system, pediatric patients, hematopoietic system, bone and soft tissues, brain, and digestive system—and specialists in fertility, nursing, and pharmacology. Moreover, for the preparation of this guideline, support was obtained from the 2016 project entitled “Establishment of Integrated Platform for Drastic Advancement in Fertility Preservation Cancer Therapy (representative investigator: Yutaka Osuga) performed by the Japan Agency for Medical Research and Development.

**Table 1:** Definitions of recommendation grades

Recommendation grade	Definition
A	Based on full scientific evidence, the approach is strongly recommended
B	Based on scientific evidence, the approach is recommended
C1	Despite the presence of limited scientific evidence, the approach is recommended
C2	Because of the paucity of scientific evidence, the approach is not recommended
D	Based on scientific evidence for its non-efficacy or harm(s), the approach is not recommended

## Chapter 1: Gynecologic cancers

### General remarks

Typical cancers of the female genital organs include uterine cervical cancer, endometrial cancer, and ovarian tumor. Of these, cervical cancer most often affects women under 40 years of age, although in recent years, endometrial cancer and ovarian tumor have become more prevalent among younger adults. Because cancers of the female genital organs affect the main organs involved in fertility, treatments for these cancers are likely to lead directly to the loss of fertility. Therefore, measures to preserve fertility in patients with these types of cancers are particularly important.

#### Clinical Question 1

Which patients with cervical cancer are eligible for conservative surgery (trachelectomy)?

In recent years, the number of young women with cervical cancer in whom fertility preservation should be considered has been increasing because the age of onset of cervical cancer has been decreasing and the tendency to marry and have a child at a later age has been increasing. According to the Patient Annual Report for Fiscal Year 2010 issued by the Japan Society of Obstetrics and Gynecology (JSOG) Gynecological Oncology Committee, the number of patients with stage IA1 to IB1 cervical cancer who were younger than 40 years of age was estimated to be 1105, and these patients were estimated to account for 33% of all patients with cervical cancer at these stages [2]. To answer this clinical question (CQ), we discussed which patients with cervical cancer are eligible for conservative surgery (trachelectomy) on the assumption that the pathological diagnosis and staging of the malignancy are accurate.

The clinical stages referred to in this CQ were assigned according to the JSOG 2011/International Federation of Gynecology and Obstetrics (FIGO) 2008 cervical cancer staging criteria, which are presented in Japanese in the General Rules for Clinical and Pathological Management of Uterine Cervical Cancer, 3rd edition [3].

##### Recommendation 1

1-1. Patients with squamous cell carcinoma or adenocarcinoma may be considered as primary candidates for conservative surgery (trachelectomy). (Recommendation Grade C1).

1-2. Conservative surgery (trachelectomy) may be considered for tumors with a diameter equal to or less than 2 cm that are localized in the uterine cervix. (Recommendation Grade C1).

#### Clinical Question 2

**Fig. 1:**
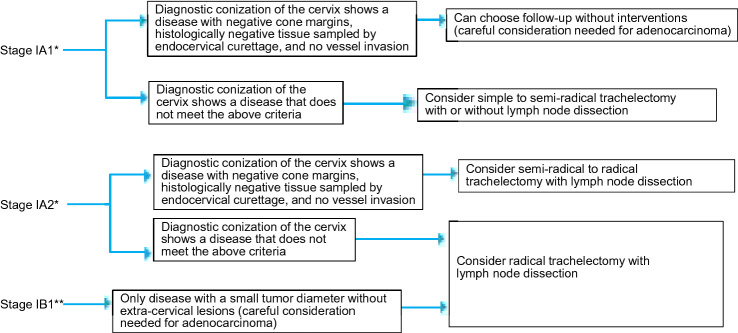
Algorithm for fertility preservation in women with cervical cancer (Chapter 1, Clinical Question 2). *Only if the disease is diagnosed by cervical conization. **Some patients with stage IIA1 disease and limited vaginal wall involvement may be considered eligible for conservative surgery (trachelectomy)

Which surgical procedures are recommended to preserve fertility in patients with cervical cancer (Fig. 1)?

When considering this CQ, we discussed the optimal surgical procedures for preserving fertility in patients with cervical cancer by stage and by histological type of the disease.

##### Recommendation 2

2-1. Disease at stage IA1 or less that is confirmed at conization to have no vessel invasion, negative cone margins bilaterally, and negative histology of endocervical curettage may require no further interventions. (Recommendation Grade C1).

2-2. Semi-radical trachelectomy plus pelvic lymph node dissection or radical trachelectomy may be considered depending on the tumor interstitial invasion depth, presence or absence of vessel invasion, and tumor diameter. (Recommendation Grade C1).

#### Clinical Question 3

**Fig. 2:**
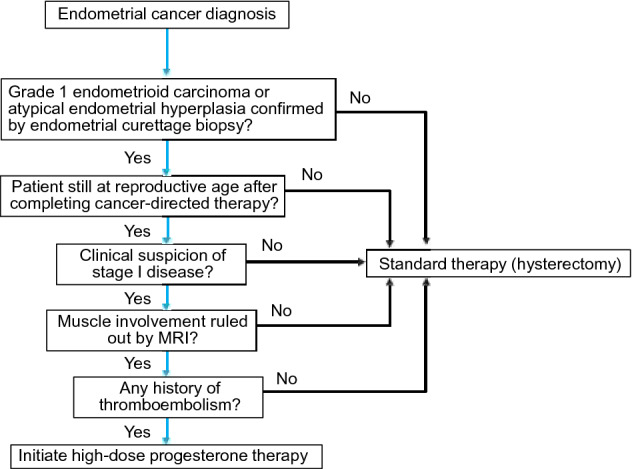
Algorithm for selecting high-dose progesterone therapy for endometrial cancer (Chapter 1, Clinical Question 3). *MRI* magnetic resonance tomography

Which patients with endometrial cancer are eligible for fertility preservation (high-dose progesterone therapy) (Fig. 2)?

In Japan, patients with endometrial cancer under 40 years of age account for 5.1% of all patients with this malignancy, but the percentage has been increasing steadily in association with a considerable increase in the number of patients with endometrial cancer. The standard therapy for endometrial cancer is hysterectomy, and an increasing number of patients undergo fertility preservation. Hence, we conducted a survey to ascertain the current consensus on the indications for such therapy.

##### Recommendation 3

Fertility preservation may be considered for highly differentiated endometrioid carcinoma (Grade 1) or atypical endometrial hyperplasia that is probably localized within the endometrium. (Recommendation Grade C1).

#### Clinical Question 4

**Fig. 3:**
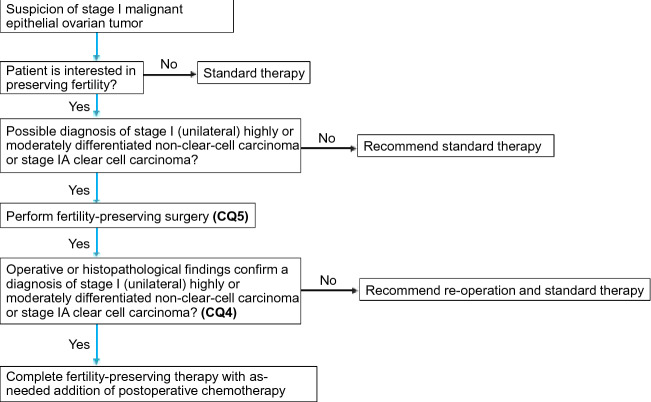
Algorithm for fertility preservation in women with ovarian cancer (Chapter 1, Clinical Questions 4, 5)

Which patients with malignant ovarian tumors are eligible for fertility preservation (Fig. 3)?

In Japan, the number of patients with newly diagnosed malignant ovarian tumors has been increasing and is expected to exceed 10,000 per year in the near future. At the same time, the tendency for women to marry later has also been increasing, leading to more pregnancies at higher ages. Therefore, an increasing number of patients with malignant ovarian tumors will be interested in fertility preservation. To answer this CQ, we discussed the indications for fertility preservation in women with malignant ovarian tumors.

##### Recommendation 4

4-1. Fertility preservation may be considered for stage IA epithelial malignant ovarian tumors, stage IC (unilateral) Grade 1 or 2 non-clear-cell carcinoma, and stage IA clear cell carcinoma. (Recommendation Grade C1).

4-2. Fertility preservation may be considered for stage I–III borderline epithelial ovarian tumors. (Recommendation Grade C1).

4-3. Fertility preservation is recommended for stage I to IV ovarian germ cell tumors. (Recommendation Grade B).

4-4. Fertility preservation may be considered for stage IA ovarian stromal cell tumor. (Recommendation Grade C1).

#### Clinical Question 5

Which surgical procedures are recommended to preserve fertility in patients with malignant ovarian tumors (Fig. 3)?

We discussed this CQ to determine the optimal surgical procedures to preserve fertility in patients with malignant ovarian tumors.

##### Recommendation 5

5-1. For epithelial malignant ovarian tumors, adnexectomy of the affected side plus omentectomy, intraperitoneal cytology, and pelvic/paraaortic lymph node dissection (biopsy) with or without contralateral ovarian biopsy and with or without biopsy of intraperitoneal lesions may be considered. (Recommendation Grade C1).

5-2. For borderline epithelial ovarian tumors, adnexectomy of the affected side plus omentectomy, intraperitoneal cytology, and intraperitoneal observation may be considered. (Recommendation Grade C1).

5-3. For ovarian germ cell tumors, adnexectomy of the affected side plus omentectomy, intraperitoneal cytology, and intraperitoneal observation is recommended. (Recommendation Grade B).

5-4. For ovarian stromal cell tumors, adnexectomy of the affected side plus omentectomy, intraperitoneal cytology, and intraperitoneal observation may be considered. (Recommendation Grade C1).

#### Clinical Question 6

What reproductive support should be provided to patients with gynecologic malignancy who have undergone fertility preservation?

Fertility preservation for gynecologic malignancy can be surgical or medical. Because gynecologic malignancy affects the organs involved in fertility, a number of points had to be considered in addressing this CQ. For example, questions arose such as whether patients undergoing surgery for cervical cancer require any assisted reproductive technology (ART) intervention because of the altered structure of their uterus or whether patients receiving medical therapy for endometrial cancer require any ART intervention to enable early pregnancy. If these questions were left unanswered, the significance of fertility preservation per se might be doubted. The term reproductive support used in the recommendations below means a broad concept that involves a wide range of attempts to become pregnant, ranging from the pre-interventional stage (e.g., self-timing) to simpler and more advanced infertility interventions.

##### Recommendation 6

6-1. Artificial insemination or ex vivo fertilization may be considered as reproductive support for patients with cervical cancer who have undergone radical trachelectomy as fertility preservation surgery. (Recommendation Grade C1).

6-2. Artificial insemination or ex vivo fertilization may be considered as reproductive support for patients with endometrial cancer to enable early pregnancy. (Recommendation Grade C1).

6-3. Ex vivo fertilization may be considered as reproductive support for patients with ovarian cancer who have been fully informed about the risk of malignant dissemination or metastasis associated with oocyte harvesting. (Recommendation Grade C1).

## Chapter 2: Breast cancer

### General remarks

**Fig. 4:**
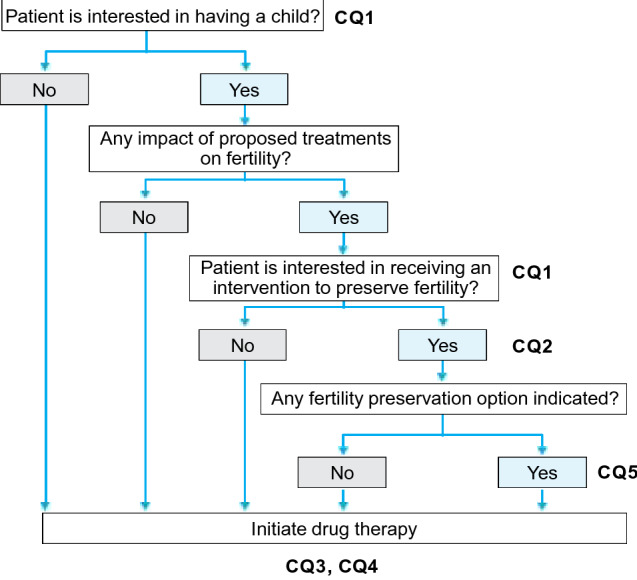
Algorithm for fertility preservation before initiation of drug therapy for patients with breast cancer (Chapter 2, Clinical Questions 1–5)

At the time of diagnosis of breast cancer, young female patients express an interest in having a child, partially because of the tendency for women to become pregnant at a later age. The probability of long-term survival of breast cancer patients after treatment has increased with improved early detection of the disease and outcomes of medical therapy. However, the hormone-dependent nature of breast cancer, chemotherapy-induced impairment of ovarian function, and increasing age during long-term endocrine therapy are the main obstacles to conception and parturition in women with breast cancer. When considering the CQs, we reviewed the important points for oncologists and reproductive specialists to consider in discussing conception and fertility preservation with women with breast cancer (Fig. 4).

#### Clinical Question 1

Which patients with breast cancer are eligible for fertility preservation?

Treatments for breast cancer should not be uniform but should be individualized by assessing the recurrence risk estimated from the clinical stage and subtype of the cancer, expected response to treatment (responsiveness to drugs), and potential adverse reactions to treatments. Because about 70% of primary breast cancers are estrogen receptor-positive, hormone-sensitive tumors, posttreatment changes in the hormonal environment associated with conception or fertility preservation or both may have negative impacts on the prognosis of breast cancer. To answer this CQ, we reviewed the possibility of pregnancy after breast cancer treatment and the indications for fertility preservation among women with breast cancer from the viewpoint of the impact of treatment and fertility preservation on the prognosis of this malignancy. Our aim was to prevent suboptimal reproductive counselling that might unnecessarily discourage patients from becoming pregnant or make them have excessive expectations about any future pregnancy.

##### Recommendation 1

1-1. Fertility preservation may be considered in patients with stage 0 to III breast cancer who are to receive standard treatment and likely to survive for a long time after treatment. (Recommendation Grade C1).

1-2. Patients with stage IV breast cancer with distant metastasis or recurrent breast cancer are not eligible for fertility preservation. (Recommendation Grade D).

#### Clinical Question 2

Is delayed initiation of chemotherapy permitted in patients with breast cancer who are interested in fertility preservation?

When patients with breast cancer receive any ART fertility preservation intervention, initiation of chemotherapy may need to be delayed to enable oocyte retrieval after ovarian stimulation. Hence, we discussed how long chemotherapy can be delayed for fertility preservation.

##### Recommendation 2

2-1. For fertility preservation, initiation of adjuvant chemotherapy may be delayed until 12 weeks postoperatively. However, some studies have suggested that delaying chemotherapy by more than 5 weeks has a negative impact on the prognosis of breast cancer. Therefore, any fertility preservation intervention before postoperative chemotherapy should be performed as quickly as possible. (Recommendation Grade C1).

2-2. Delaying initiation of neoadjuvant chemotherapy for fertility preservation is not permitted, because its safety has not been established. Any fertility preservation intervention before neoadjuvant chemotherapy should be performed as quickly as possible to avoid delays in initiating the planned chemotherapy. (Recommendation Grade C2).

#### Clinical Question 3

From the aspect of the prognosis of malignancy, when can a woman with stage I–III breast cancer who is interested in becoming pregnant consider pregnancy after completing cancer-directed therapy?

We considered both (1) the effect of conception on the prognosis (e.g., recurrence risk) of breast cancer and (2) the embryofetal toxicity (e.g., teratogenicity) of the cancer-directed therapy. In the recommendation below, we address this question from the former aspect; the latter aspect is addressed in the subsequent CQ.

Because a woman with breast cancer becomes older while receiving long-term endocrine therapy, she may have a reduced possibility of becoming pregnant if she waits until completion of endocrine therapy. Hence, we also addressed the question whether a woman with breast cancer can interrupt endocrine therapy to attempt to become pregnant.

##### Recommendation 3

Women with breast cancer who have completed standard treatment comprising surgery, radiotherapy, chemotherapy, and/or endocrine therapy may consider becoming pregnant, because their pregnancy will have little adverse effect on the prognosis of their breast cancer. The acceptable timing for initiating attempts to become pregnant may be individualized according to the subtype and recurrence risk of breast cancer. (Recommendation Grade C1).

When top priority is given to reducing the recurrence risk and breast cancer mortality, it is recommended to administer adjuvant hormone therapy or oral tamoxifen for 5 years or longer. With regard to the recurrence risk if oral medication is suspended in women who wish to have a baby, the results of an ongoing prospective, global study (POSITIVE study) by the International Breast Cancer Study Group (IBCSG) are awaited [13].

#### Clinical Question 4

From the aspect of embryofetal toxicity (e.g., teratogenicity) of medical treatment and radiotherapy for breast cancer, when can a woman with breast cancer who is interested in becoming pregnant consider pregnancy after completing cancer-directed therapy?

In this CQ, we considered the question of the timing of pregnancy after cancer-directed therapy from the aspect of embryofetal toxicity (e.g., teratogenicity) of cancer treatment.

##### Recommendation 4

After the end of cancer-directed therapy, a washout period or period of contraception of adequate duration should be adhered to, depending on the teratogenic potential of the drug(s) used. The acceptable timing of initiating attempts to become pregnant after undergoing radiotherapy may be determined on the basis of the estimated recurrence risk and the medical treatment plan. (Recommendation Grade C1).

#### Clinical Question 5

Which fertility preservation options can be offered to a woman with breast cancer who is interested in becoming pregnant?

Anticancer drugs used to treat female cancers (e.g., alkylating agents) are known to be gonadotoxic and to reduce the ovarian reserve. The incidence of amenorrhea (anovulation) for 3 months or longer within 1 year after treatment for breast cancer varies across drugs and regimens used and ranges from 20 to 100% [4].

The American Society of Clinical Oncology (ASCO) Clinical Practice Guideline Update on Fertility Preservation for Patients With Cancer states that 30–70% of women with breast cancer who are under 40 years of age develop chemotherapy-induced amenorrhea after receiving 4 cycles of doxorubicin plus cyclophosphamide combined with a taxane, a finding that classifies this chemotherapy regimen as having an intermediate infertility risk [5]. These data predict that young women with breast cancer will develop refractory infertility due to premature ovarian failure immediately after receiving treatment with this standard regimen. The time lag after the end of cancer treatment until permission can be given to attempt pregnancy or until attempting pregnancy is deemed socially acceptable carries the risk of a further reduction of fertility with aging [6, 7]. Taking all these risks into account, it seems very important to educate women with breast cancer who are interested in having a child about the possibility of reduced fertility resulting from cancer treatment and to counsel them regarding fertility preservation options.

When considering this CQ, we intended to assist health care providers in providing women with breast cancer with the most current information on embryo (fertilized oocyte) cryopreservation, unfertilized oocyte cryopreservation, ovarian tissue cryopreservation, and ovarian suppression with a gonadotropin-releasing hormone (GnRH) agonist as fertility preservation options that can be used before initiation of breast cancer treatment.

##### Recommendation 5

5-1. Embryo (fertilized oocyte) cryopreservation is recommended for female patients who have a male partner. (Recommendation Grade B).

5-2. Unfertilized oocyte cryopreservation may be considered for female patients who do not have a male partner. (Recommendation Grade C1).

5-3. Ovarian tissue cryopreservation, although it remains experimental, may be considered in centers with the necessary expertise for female patients who do or do not have a male partner if there is a need for urgent cancer-directed therapy that may limit the time available for embryo (fertilized oocyte) or unfertilized oocyte cryopreservation or if induction of ovulation for oocyte harvesting is difficult (e.g., in prepubertal females). (Recommendation Grade C1).

5-4. Use of GnRH agonists for the purpose of fertility preservation is not recommended. (Recommendation Grade C2).

## Chapter 3: Urologic cancers

### General remarks

**Fig. 5:**
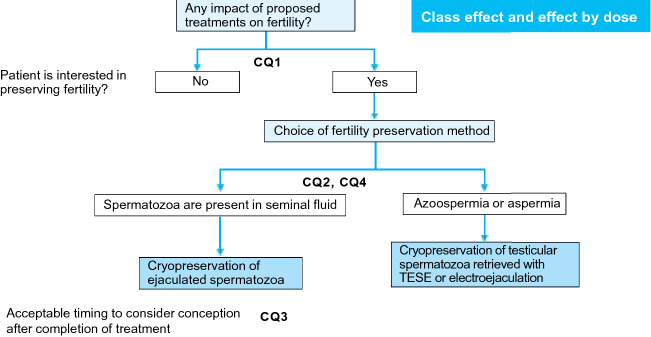
Algorithm for fertility preservation before initiation of chemotherapy in patients with a testicular tumor (Chapter 3, Clinical Questions 1–4). *TESE* testicular sperm extraction

Typical malignancies that affect the urological system or male genital organs include renal cancer, urothelial cancer, prostate cancer, and testicular tumor. Of these malignancies, testicular tumor frequently affects men aged 20–49 years, i.e., men in whom fertility preservation is most relevant. Renal, urothelial, and prostate cancers have higher incidence rates than testicular tumor but generally affect individuals aged 60 years and above. Of note, even middle-aged or older male patients may have an interest in having a child, and such patients should be counselled regarding fertility preservation. Renal and urothelial cancers less frequently affect women aged 20–49 years. To answer the following CQs, we reviewed the epidemiology, methods of treatment, and prognosis of individual types of urological malignancies (Fig. 5).

#### Clinical Question 1

Which patients with urological malignancy should be counselled regarding fertility preservation interventions?

The therapeutic outcome for urological malignancies in young adults has been improving with the advancement of treatment options. In particular, in many patients, testicular tumors have become curable with chemotherapy and surgery. This development has increased the importance of fertility preservation to maintain the patient’s quality of life (QOL) after cancer-directed therapy. Nevertheless, in practice, patients with testicular tumors appear not to be sufficiently counselled regarding fertility preservation or provided with optimal fertility preservation measures; this is even more the case in patients with renal and bladder cancers, which occur also at young ages. Of primary importance is that treating oncologists recognize which patients with urological malignancies are eligible for fertility preservation and counsel these patients regarding this issue. Of importance is also to establish a nationwide system that enables fertility preservation interventions, including sperm cryopreservation, to be implemented without the need to delay initiation of cancer-directed therapy. To answer this CQ, we considered which patients with urological malignancies are eligible for fertility preservation.

##### Recommendation 1

If a patient scheduled to receive a treatment that is likely to cause infertility is interested in fertility preservation, it is recommended to counsel the patient regarding fertility preservation interventions before initiation of cancer-directed therapy. (Recommendation Grade B).

#### Clinical Question 2

Is delayed initiation of cancer-director therapy permitted in patients with urological malignancies who are interested in fertility preservation to afford them the opportunity to receive a fertility preservation intervention before starting cancer treatment?

Testicular tumor frequently affects young and middle-aged men, and in many patients even advanced disease is curable with chemotherapy and surgery. Patients with testicular tumor often have impaired spermatogenesis before initiation of treatment, and cisplatin impairs sperm production in a dose-dependent manner. To respond to this CQ, we addressed the optimal timing of sperm cryopreservation and the acceptability of delayed initiation of cancer treatment and thereby focused on testicular tumor.

##### Recommendation 2

The delay in initiating cancer-directed therapy to enable provision of a fertility preservation intervention should be minimized. Because some advanced testicular tumors require urgent medical therapy, the acceptability of delaying initiation of treatment may be determined on a case-by-case basis. (Recommendation Grade C1).

#### Clinical Question 3

When can a patient with a urological malignancy who is interested in having a child consider pregnancy after completing cancer-directed therapy?

The earlier diagnosis and advancement of therapy have led to improved therapeutic outcomes for urological malignancies. In particular, in many patients, testicular tumor has become curable with chemotherapy and surgery. Consequently, to improve patients’ QOL, it is important to provide appropriate information to patients with urological malignancies who hope to have a child after completing cancer treatment. We addressed this issue in this CQ.

##### Recommendation 3

3-1. In male patients, sperm cryopreservation before initiation of cancer treatment can enable later intracytoplasmic sperm injection at any time. (Recommendation Grade B).

3-2. Contraception for an appropriate period of time may be considered in patients who have used any drug that is teratogenic or does not have an established embryofetal safety. (Recommendation Grade C1).

#### Clinical Question 4

Which fertility preservation options are recommended for patients with urological malignancy who are interested in having a child?

The advancement of multi-modality therapy for urological malignancies has led to an increased probability of cure in patients at reproductive age and increased the needs for preserving patient fertility as one of the measures for improving QOL. In this CQ, we considered the current options for fertility preservation in patients with urological malignancy who are at reproductive age.

##### Recommendation 4

4-1. Sperm cryopreservation is recommended in male patients. (Recommendation Grade B).

4-2. If spermatozoa are absent in ejaculated seminal fluid, harvesting and cryopreservation of testicular spermatozoa may also be considered. (Recommendation Grade C1).

4-3. Use of GnRH agonists for the purpose of fertility preservation is not recommended in patients with testicular tumor. (Recommendation Grade D).

## Chapter 4: Pediatric cancers

### General remarks

**Fig. 6:**
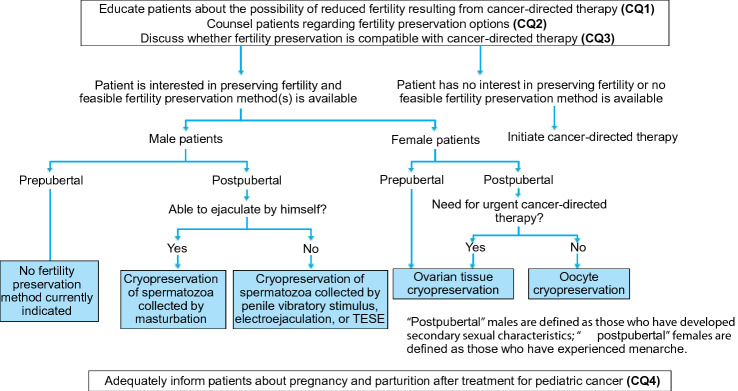
Algorithm for fertility preservation in pediatric cancer patients (Chapter 4, Clinical Questions 1–4). *TESE* testicular sperm extraction

Therapeutic outcomes for pediatric cancers have improved considerably over the past several decades. However, long-term complications such as reduced fertility have raised significant concerns for patients cured of pediatric cancer. This chapter first explains the ethical considerations that must specifically be taken into account in treating pediatric patients (Fig. 6). When considering the subsequent CQs, we aimed to provide recommendations that enable oncologists caring for pediatric cancer patients to properly assess the risk of reduced fertility after cancer treatment within the short time from the diagnosis until the initiation of treatment and to implement a fertility preservation intervention in cooperation with reproductive medicine specialists.

### Ethical considerations specific for pediatric patients

When counselling pediatric patients regarding fertility preservation and/or providing them with a fertility preservation intervention, health care providers must consider ethical considerations that are specific to this population. Because there is currently no guidance on informed consent/assent in pediatric practice in Japan, below we discuss the ethical considerations specific for pediatric patients that must be taken into account in dealing with fertility preservation issues in pediatric cancer patients by referring to the American Academy of Pediatrics (AAP) Guidelines for the Ethical Conduct of Studies to Evaluate Drugs in Pediatric Populations [8], the Guidance on Clinical Investigation of Medicinal Products in the Pediatric Population [9], and the Ethical Guidelines for Medical and Health Research Involving Human Subjects [10].

Assent, a concept advocated by the AAP in 1995, means active agreement to undergo any medical intervention by a minor who is informed to the fullest extent that their age permits [8]. The assent process consists of the following 4 steps: (1) the patient’s understanding of his/her condition to the fullest extent that his/her age permits; (2) explanation to the patient about expected outcomes of the diagnostic or therapeutic intervention proposed and their significance; (3) assessment of the level of the patient’s understanding of his/her condition and exploration of factors that affect the patient’s decision making; and iv) expression of the patient’s will whether or not to agree to undergo the medical intervention proposed. Thus, the assent process does not merely aim to receive agreement from a pediatric patient but, more importantly, to receive agreement from a pediatric patient that is given from his/her own will after being informed to the fullest extent that his/her age permits and understanding the information. Through these steps, pediatric patients develop an ability to understand their conditions and to make decisions by themselves by considering their conditions. The AAP recommends that for decision making in pediatric practice, assent should be received from pediatric patients who have reached an intellectual age of 7 years or above. The APP also recommends that before giving any intervention to adolescents who have sufficient ability to understand, their informed consent should be obtained in addition to their parents’ consent.

The Guidance on Clinical Investigation of Medicinal Products in the Pediatric Population [9] recommends that written assent to participate in a clinical study should be received from pediatric patients aged 12 years or older. Regarding the ages of pediatric patients in whom assent generally applies, the Guidance also recommends that all study participants should be informed to the fullest extent possible about the study in language and terms that they are able to understand. In the Questions and Answers on this Guidance, the Evaluation and Licensing Division of the Pharmaceutical and Medical Safety Bureau of the Ministry of Health, Labour and Welfare expresses its opinion that those aged 7 years or above are able to understand a simple explanation [11].

In Japan, the Ethical Guidelines for Medical and Health Research Involving Human Subjects [10] also make recommendations on informed consent and assent from minors. According to the recommendations, minors who have completed compulsory education or are aged 16 years or above should be regarded as having sufficient ability to decide whether or not to participate in research and as qualified to give informed consent and the best effort must be made to receive assent from minors who have not completed compulsory education and are aged under 16 years.

Based on these guidance/guideline recommendations, before providing any fertility preservation intervention to pediatric patients, it is desirable to (1) obtain informed consent from the patients and their parents if the patients have completed compulsory education or are aged 16 years or above and are considered to have sufficient ability to make decisions and (2) inform the patients in an age-specific manner and to receive their assent, in addition to their parents’ informed consent, if the patients have not completed compulsory education and are aged under 16 years.

#### Clinical Question 1

Which pediatric cancer patients are eligible for fertility preservation?

To answer this question, we also considered the aspect of the level of understanding of the patient and of the patient’s parents, as well as the ethical background of fertility preservation in pediatric patients. For this purpose, we first considered the infertility problems encountered by survivors of pediatric cancers [5, 12]. Here, we discussed the indications for fertility preservation in pediatric cancer patients by considering their ages and whether (1) planned chemo- or radiotherapy would impair or completely abolish gonadal function, (2) survival is highly probable after cancer treatment, (3) the patients have enough time and physical strength to receive any fertility preservation intervention, and (4) the patient and/or the patient’s parents can fully understand that ovarian tissue cryopreservation remains experimental.

The solid tumors we reviewed included brain tumors (e.g., medulloblastoma and germ cell tumor), neuroblastoma, non-central nervous system germ cell tumor, rhabdomyosarcoma, osteosarcoma, Ewing sarcoma, retinoblastoma, and non-rhabdomyosarcoma soft tissue tumor. Fertility preservation is indicated for patients who require a treatment approach for these types of pediatric cancers that is likely to cause infertility, under consideration of the reported outcomes and probability of survival after treatment, and who have enough time and physical strength to undergo a fertility preservation intervention before initiation of the treatment.

Standard chemotherapy for pediatric hematologic malignancies (e.g., leukemias and lymphomas) reduces fertility in less than 20% of patients. However, fertility preservation is indicated for pediatric patients who are to receive any procarbazine-containing chemotherapy regimen for Hodgkin’s disease or to undergo radiotherapy directed toward the gonadal system or pelvis or hematopoietic cell transplant (HCT). Patients undergoing HCT frequently experience an irreversible reduction of fertility, although the risk of infertility varies across the conditioning regimens performed before HCT. Therefore, postpubertal pediatric cancer patients and their parents should be counselled to inform them about the concept of fertility preservation.

We also specifically reviewed fertility preservation options for female and male pediatric cancer patients. In prepubertal female patients, ovarian tissue cryopreservation may be considered and should be proposed, even though this technique remains experimental. The Edinburgh criteria [4] should be followed to determine which female patients are eligible for this fertility preservation option. Ovarian tissue cryopreservation is also an option for postpubertal female patients if cancer treatment needs to be initiated quickly, and it may be chosen after full counselling of the patients and their family. Another option for postpubertal female patients is unfertilized oocyte cryopreservation.

In male pediatric cancer patients, no technically established fertility preservation options are available for prepubertal patients. However, all postpubertal patients who are interested in having a child should preferably be offered counselling on fertility preservation, more concretely, on sperm cryopreservation before initiation of cancer treatment.

##### Recommendation 1

Regardless of the type of cancer, pediatric cancer patients scheduled to receive any treatment that is likely to cause infertility should be considered eligible for fertility preservation, whereby the treatment regimen used and the probability of survival after cancer treatment should also be taken into account. (Recommendation Grade B).

#### Clinical Question 2

Which fertility preservation options can be offered to pediatric cancer patients?

The therapeutic outcomes for pediatric cancers have been improving such that the 5-year survival rate is reported to have reached 80% in all pediatric cancer patients. This higher survival rate has increased the importance of reducing the risk of long-term complications of cancer treatment and helping survivors achieve better QOL. Fertility preservation is an important challenge, among others. In this CQ, we conducted an evidence-based assessment and review of fertility preservation options that can be offered to pediatric cancer patients.

##### Recommendation 2

In pediatric cancer, different fertility preservation options are indicated in prepubertal versus postpubertal patients and male versus female patients. Therefore, the recommendation is subdivided according to the individual populations.

2-1. Unfertilized oocyte cryopreservation may be considered in postpubertal female patients. (Recommendation Grade C1).

2-2. Ovarian tissue cryopreservation is the only fertility preservation option for prepubertal female patients. It may also be considered for postpubertal female patients if there is a need for urgent cancer treatment. This technique still remains experimental, but it may be performed in the context of clinical trials in centers with the necessary expertise. (Recommendation Grade C1).

2-3. Ovarian transposition (oophoropexy) is recommended in both prepubertal and postpubertal female patients if pelvic irradiation is performed. (Recommendation Grade B).

2-4. Sperm cryopreservation is recommended in postpubertal male patients. (Recommendation Grade B).

2-5. No fertility preservation options are currently indicated for prepubertal male patients. (No Recommendation Grade).

#### Clinical Question 3

Can treatment of pediatric cancers be delayed to allow fertility preservation?

Pediatric cancer patients can receive a fertility preservation intervention only if the intervention will have no adverse effect on the prognosis of the cancer. However, the intervention may require the initiation of cancer-directed therapy to be delayed. Because pediatric cancer is generally a rapidly progressing disease, it is desirable to initiate treatment quickly after diagnosis. Hence, when considering this CQ, we discussed whether treatment of pediatric cancer can be delayed to enable fertility preservation.

##### Recommendation 3

Delaying cancer-directed therapy may be considered only if the delay will have no adverse effect on the prognosis of the cancer. (Recommendation Grade C1).

#### Clinical Question 4

How should pediatric cancer patients be counseled regarding pregnancy and parturition after completion of treatment?

With the improvement of therapeutic outcomes, some pediatric cancer patients have achieved long-term survival, and survivors of pediatric cancer may become able to have a child. Both patients and health care providers have become more aware of the risks of infertility and premature ovarian failure associated with cancer treatment and of the maternal and offspring health status among cancer survivors who maintain fertility. Evidence shows that cancer treatment is unlikely to cause an increased frequency of congenital anomalies in offspring, but there are some reports of an increased risk of premature parturition if the mother has previously received cancer treatment. We discussed the risks for pregnancy and parturition in survivors of pediatric cancers by considering the results of large overseas cohort studies.

##### Recommendation 4

4-1. Survivors of pediatric cancers should be informed that there is no direct evidence for a significant increase in the risk of congenital anomalies in children born to parents who have previously received cancer treatment. (Recommendation Grade B).

4-2. Female survivors of pediatric cancers should be informed that risks during pregnancy and at parturition are different across different types of cancer, ages at treatment, and treatment regimens used. (Recommendation Grade B).

4-3. Female patients who have undergone abdominal/pelvic irradiation should be informed that they have to be closely monitored during pregnancy until parturition to prevent spontaneous abortions and premature deliveries. (Recommendation Grade B).

## Chapter 5: Hematologic cancers

### General remarks

**Fig. 7:**
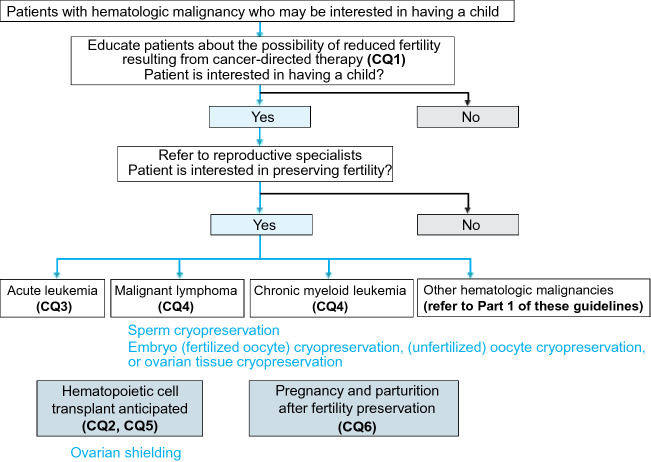
Algorithm for fertility preservation in patients with hematologic malignancy (Chapter 5, Clinical Questions 1–6)

The hematologic malignancies that rarely occur in young individuals are referred to only in Part 1 of this guideline [1] and were not addressed in the following CQs. Instead, in Clinical Questions 1 through 5, we considered the common effects on fertility of treatments for hematologic malignancies (chemotherapy, radiotherapy, molecular targeting therapy, and HCT) and the fertility preservation methods available for patients with these malignancies. In Clinical Question 6, we reviewed whether fertility preservation in a patient with a hematologic malignancy can have any adverse effects on offspring if the patient or the patient’s partner becomes pregnant after cancer treatment (Fig. 7).

#### Clinical Question 1

Which patients with hematologic malignancies are eligible for fertility preservation?

Treatments for hematologic malignancies include chemotherapy, radiotherapy, molecular targeting therapy, and HCT. These treatments can affect spermatogenesis and menstruation. Standard chemotherapy regimens for acute leukemia, non-Hodgkin’s lymphoma, and Hodgkin’s disease have a low risk for infertility, whereas procarbazine-containing chemotherapy regimens for Hodgkin’s disease, gonadal/pelvic irradiation, and HCT conditioning considerably reduce fertility in both male and female patients. Thus, a hematologic malignancy may progress to a stage that requires treatment that will have a marked effect on fertility. Hence, we discussed which patients with hematologic malignancies are eligible for fertility preservation.

##### Recommendation 1

1-1. Regardless of the type of hematologic malignancy, whenever possible fertility preservation should be considered before initiation of cancer-directed therapy. (Recommendation Grade C1).

1-2. If fertility preservation is not feasible before initiation of cancer treatment, it may be considered again when any change is made to the treatment strategy. (Recommendation Grade C1).

#### Clinical Question 2

How should patients with hematologic malignancies be counselled regarding the impact of HCT on their fertility?

HCT conditioning with high-dose chemotherapy and/or total body irradiation has a greater impact on fertility than other cancer treatments. With the improved outcomes of HCT, the number of long-term survivors of hematologic malignancies has been increasing. Because loss of fertility would markedly impair survivors’ QOL, we discussed how patients with hematologic malignancies should be counselled regarding the impact of HCT on their fertility.

##### Recommendation 2

2-1. Both male and female patients should be informed that HCT often leads to irreversible impairment of fertility. (Recommendation Grade B).

2-2. Patients may be informed that it is uncertain whether their fertility can be better preserved with reduced intensity conditioning before HCT. (Recommendation Grade C1).

#### Clinical Question 3

Which fertility preservation methods can be recommended for patients with acute leukemia who are interested in having a child?

Acute leukemia frequently affects young individuals. The first treatment goal is to achieve complete remission, which requires urgent intensive combination chemotherapy with cytotoxic agents. Female patients in particular have no time to spare for a fertility preservation intervention before initiation of cancer treatment. We discussed which fertility preservation methods can be recommended for patients with acute leukemia who will potentially receive treatment that is likely to reduce their fertility.

##### Recommendation 3

3-1. Although initial standard chemotherapies for hematologic malignancies are usually not highly gonadotoxic, in centers where they are available reproductive specialists should be consulted promptly. (Recommendation Grade B).

3-2. Embryo (fertilized oocyte) cryopreservation is recommended for female patients who have a male partner. (Recommendation Grade B).

3-3. Unfertilized oocyte cryopreservation may be considered for female patients who do not have a male partner. (Recommendation Grade C1).

3-4. In general, ovarian tissue cryopreservation is not recommended for female patients who do or do not have a male partner because of the risk of leukemic cells contaminating tissue; however, this technique is currently performed in some centers in an experimental manner with the expectation that future developments may make it a feasible procedure. (Recommendation Grade C2).

3-5. Use of GnRH agonists may be considered for the purpose of controlling menstruation but is not recommended for the purpose of fertility preservation. (Recommendation Grade C2).

3-6. Sperm cryopreservation is recommended for postpubertal male patients and, whenever possible, should be performed before initiation of treatment. (Recommendation Grade B).

#### Clinical Question 4

Which fertility preservation methods can be recommended for patients with other hematologic malignancies who are interested in having a child?

In this CQ, we focused on lymphoma and chronic myeloid leukemia, which—like acute leukemia—often affect individuals at reproductive age. Other hematologic malignancies typically occur at older ages, and little information is available on the impacts on fertility of the malignancies and the associated treatments. Therefore, these malignancies were not addressed in this CQ, but they are briefly reviewed in Part 1 of this guideline.

Patients scheduled to receive lymphoma treatments, which have a low risk for infertility (refer to Chapter 5, CQ1), are likely to maintain fertility after treatment and should not unnecessarily delay initiation of cancer treatment. However, lymphoma may potentially progress to a stage that requires treatment with a high risk for infertility (e.g., alkylating agent-based regimens and HCT).

Imatinib, a drug used to treat chronic myeloid leukemia, has been shown to be teratogenic, but there is no definitive evidence for the teratogenicity of second- or later generation tyrosine kinase inhibitors or for their impacts on fertility. Because in the future, an increasing number of newer molecular targeting agents will be introduced into the market for the treatment of chronic myeloid leukemia, attention should be paid to the individual teratogenicity and impacts on fertility of these agents.

Considering these circumstances, we discussed which fertility preservation methods can be recommended for patients with lymphoma and chronic myeloid leukemia.

##### Recommendation 4

4-1. Lymphoma.

4-1-1. Embryo (fertilized oocyte) cryopreservation is recommended for female patients who have a male partner. (Recommendation Grade B).

4-1.2. Unfertilized oocyte cryopreservation may be considered for female patients who do not have a male partner. (Recommendation Grade C1).

4-1.3. Ovarian tissue cryopreservation, although it remains experimental, may be considered in centers with the necessary expertise for female patients who do or do not have a male partner if there is a need for urgent cancer-directed therapy such that there may be limited time for embryo (fertilized oocyte) or unfertilized oocyte cryopreservation or if ovulation induction is difficult for oocyte harvesting (e.g., in prepubertal female patients). (Recommendation Grade C1).

4-1-4. Use of GnRH agonists may be considered for the purpose of controlling menstruation but is not recommended for the purpose of fertility preservation. (Recommendation Grade C2).

4-1-5. Sperm cryopreservation is recommended for male patients and, whenever possible, should be performed before initiation of treatment. (Recommendation Grade B).

4-2. Chronic myeloid leukemia.

These recommendations can be made to patients who are interested in receiving a fertility preservation intervention after being informed that molecular targeting agents have uncertain impacts on fertility.

4-2-1. Embryo (fertilized oocyte) cryopreservation is recommended for female patients who have a male partner and should be performed before initiation of molecular targeting therapy. (Recommendation Grade B).

4-2-2. Before initiation of molecular targeting therapy, unfertilized oocyte cryopreservation may be considered in female patients who do not have a male partner. (Recommendation Grade C1).

4-2-3. Sperm cryopreservation is recommended for postpubertal male patients and, whenever possible, should be performed before initiation of treatment. (Recommendation Grade B).

#### Clinical Question 5

Which fertility preservation methods can be recommended for patients undergoing HCT who are interested in having a child?

HCT is likely to cause patients to lose fertility. When undergoing HCT, patients with hematologic malignancies may have already lost fertility because of the effects of the underlying malignancy or prior cancer treatments. Even patients who have not lost fertility often lose it after undergoing HCT. Some patients with acute leukemia become eligible for HCT after failure of initial induction chemotherapy. Therefore, patients with hematologic malignancies should be counselled regarding fertility preservation before initiation of treatment. In this CQ, we discussed which fertility preservation methods can be recommended for patients undergoing HCT, regardless of the type of cancer.

##### Recommendation 5

5-1. Embryo (fertilized oocyte) cryopreservation is recommended as long as this intervention does not compromise cancer treatment outcomes. (Recommendation Grade B).

5-2. Unfertilized oocyte cryopreservation may be considered as long as this intervention does not compromise cancer treatment outcomes. (Recommendation Grade C1).

5-3. Use of GnRH agonists may be considered for the purpose of controlling menstruation but is not recommended for the purpose of fertility preservation. (Recommendation Grade C2).

5-4. Sperm cryopreservation is recommended for male patients and, whenever possible, should be performed before initiation of treatment. (Recommendation Grade B).

5-5. Testicular shielding is not recommended. (Recommendation Grade C2).

#### Clinical Question 6

How should patients with hematologic malignancies be counselled regarding posttreatment pregnancy and parturition?

Recent improvements in cancer treatments and technological advancements in reproductive medicine have enabled an increasing number of patients with hematologic malignancies to conceive and give birth to a child after completing cancer treatment. However, because the anti-cancer agents used and the duration of their use, as well as recurrence risk, vary across the different types of malignancy, the question when pregnancy may be considered after completion of cancer treatment should be discussed from the aspects of both the impacts of conception on the prognosis (e.g., recurrence) of malignancy and the embryofetal toxicity of cancer treatment. In this CQ, we discussed how patients with hematologic malignancies should be counselled regarding pregnancy and parturition after completion of cancer treatment.

##### Recommendation 6

6-1. Patients should be informed that it is difficult to apply uniform criteria to determine the acceptable timing for considering pregnancy after completion of cancer treatment. (Recommendation Grade B).

6-2. Patients with hematologic malignancies should be informed that if they or their partners have become pregnant after cancer treatment it is uncertain whether the risk of congenital anomalies in their children is higher than in children of parents who did not previously receive cancer treatment. (Recommendation Grade B).

6-3. Female patients who have undergone abdominal/pelvic irradiation should be closely monitored during pregnancy until parturition to prevent spontaneous abortions and premature deliveries. (Recommendation Grade B).

## Chapter 6: Bone and soft tissue tumors

### General remarks

**Fig. 8:**
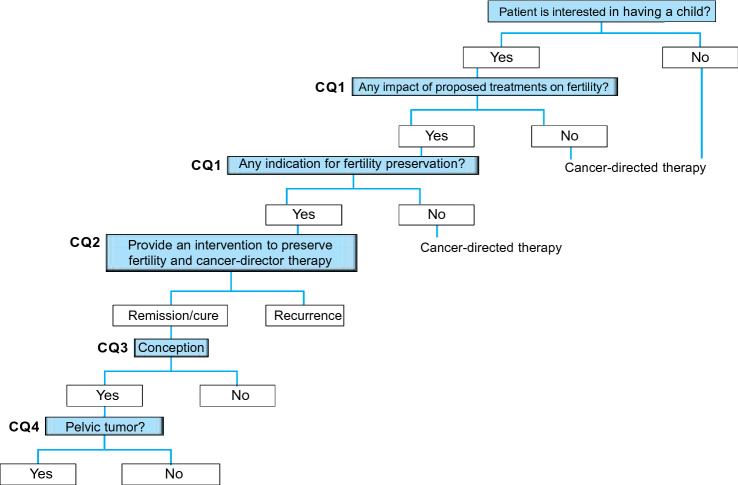
Algorithm for fertility preservation in patients with bone and soft tissue tumors (Chapter 6, Clinical Questions 1–4)

Bone and soft tissue tumors are of mesenchymal origin, and the majority of malignant bone and soft tissue tumors are sarcomas. Because bone and soft tissue sarcomas often affect children, adolescents, and young adults (aged 15–39 years), many patients with this malignancy are eligible for fertility preservation (Clinical Questions 1 through 3). Sarcomas can develop in any part of the body, but many of them occur in the pelvis and can thus affect the patient’s ability to become pregnant and give birth (Clinical Question 4). Surgical resection is the mainstay treatment for bone and soft tissue sarcomas, and chemo- and radiotherapy are also used in the adjuvant setting (Clinical Questions 1 through 3). Multi-modality therapy has considerably improved the prognosis of bone and soft tissue sarcomas and increased the number of long-term survivors of these malignancies. In treating bone and soft tissue sarcomas, it is important to enable these survivors to have the opportunity to preserve their fertility (Fig. 8).

#### Clinical Question 1

Which patients with malignant bone and soft tissue tumors are eligible for fertility preservation?

Multi-modality therapy for malignant bone and soft tissue tumors has improved patients’ therapeutic outcomes and raised the expectations for maintaining fertility after cancer treatment. In this CQ, we discussed which patients with malignant bone and soft tissue tumors are eligible for fertility preservation.

##### Recommendation 1

Patients with malignant bone and soft tissue tumors who will be at high risk for infertility, including those requiring chemotherapy and those with tumors of pelvic or retroperitoneal origin, should be considered eligible for fertility preservation, whereby the treatment regimens used and the probability of survival should be taken into account. (Recommendation Grade B).

#### Clinical Question 2

Which fertility preservation options can be offered to patients with malignant bone and soft tissue tumors?

Recent advancements in multi-modality therapy for malignant bone and soft tissue tumors have led to improved therapeutic outcomes in CAYA patients, but concerns remain about the impacts of treatments on patients’ gonadal function. We discussed which fertility preservation methods can be recommended for patients with malignant bone and soft tissue tumors who are interested in having a child after cancer treatment.

##### Recommendation 2

Different fertility preservation options are indicated for prepubertal versus postpubertal patients and male versus female patients with malignant bone and soft tissue tumors. Therefore, the recommendation is subdivided according to the individual populations.

2-1. Embryo (fertilized oocyte) cryopreservation is recommended for female patients who have a male partner if they can wait for 2 weeks or more to initiate cancer chemotherapy. (Recommendation Grade B).

2-2. Unfertilized oocyte cryopreservation may be considered for postpubertal female patients who do not have a male partner. (Recommendation Grade C1).

2-3. Ovarian tissue cryopreservation, although it remains experimental, may be considered in centers with the necessary expertise for female patients who do or do not have a male partner if there is a need for urgent cancer-directed therapy such that there may be limited time for embryo (fertilized oocyte) or unfertilized oocyte cryopreservation or if ovulation induction is difficult for oocyte harvesting (e.g., in prepubertal female patients). (Recommendation Grade C1).

2-4. Ovarian transposition (oophoropexy) is recommended in both prepubertal and postpubertal female patients when pelvic irradiation is performed. (Recommendation Grade B).

2-5. Use of GnRH agonists for the purpose of fertility preservation is not recommended. (Recommendation Grade C2).

2-6. Sperm cryopreservation is recommended for postpubertal male patients. (Recommendation Grade B).

2-7. No fertility preservation options are currently indicated for prepubertal male patients. (No Recommendation Grade).

#### Clinical Question 3

When can a patient with malignant bone and soft tissue tumors who is interested in having a child consider pregnancy after completing cancer-directed therapy?

We performed a literature review to gather information on patients with high-grade bone and soft tissue tumors who became pregnant and gave birth to a child after completing chemotherapy and discussed the acceptable timing for patients with such tumors to consider conception.

##### Recommendation 3

3-1. If any teratogenic anti-cancer drug is used, contraception may be considered until the drug and its metabolite(s) are no longer detectable in the body or until they can assume to no longer be present on the basis of the drug half-life. (Recommendation Grade C1).

3-2. In male patients, sperm cryopreservation before medical cancer treatment or total body irradiation can enable later intracytoplasmic sperm injection at any time. (Recommendation Grade B).

3-3. During the first 2 years after completion of treatment, pregnancy may be considered if patients are made fully aware of the high risk of cancer recurrence and metastasis during this time. (Recommendation Grade C1).

#### Clinical Question 4

Are patients who are treated for malignant bone and soft tissue tumors arising from the pelvis able to become pregnant and give birth to a child?

Multi-modality therapy for malignant bone and soft tissue tumors has improved the therapeutic outcomes of these tumors so that young female survivors of these tumors more often have the opportunity to become pregnant and give birth to a child. Hence, we discussed whether female patients with malignant bone and soft tissue tumors arising from the pelvis can become pregnant and give birth to a child after cancer treatment.

##### Recommendation 4

Despite the presence of various risks, female patients with pelvic malignant bone and soft tissue tumors may become pregnant and deliver babies (transvaginally) after cancer treatment. (Recommendation Grade C1).

## Chapter 7: Brain cancers

### General remarks

**Fig. 9:**
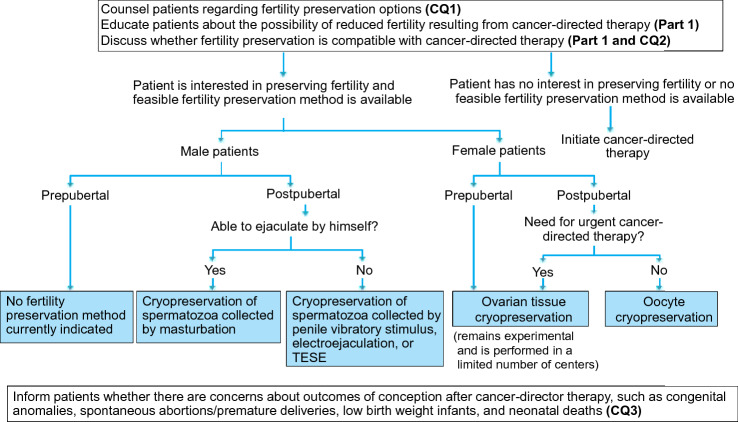
Algorithm for fertility preservation in patients with a brain tumor (Chapter 9, Clinical Questions 1–3). *TESE* testicular sperm extraction

Pediatric brain tumors for which chemo- and radiotherapy can compromise fertility preservation are medulloblastoma, pineoblastoma, and ependymoma. The pediatric brain tumors that can cause hypothalamic/pituitary dysfunction or require chemo- and radiotherapy and thus compromise fertility preservation are central nervous system germ cell tumor, optic pathway/hypothalamic pilocytic astrocytoma, and other types of astrocytoma. Craniopharyngiomas can cause hypothalamic/pituitary dysfunction or require radiotherapy that can compromise fertility preservation. Because of the prognosis, careful counselling regarding fertility preservation is needed in patients with diffuse intrinsic pontine glioma, thalamic astrocytoma, and some embryonic tumors (embryonal tumor with multilayered rosettes, embryonal tumor with abundant neuropil and true rosettes, atypical teratoid rhabdoid tumor, and choroid plexus carcinoma). Unlike pediatric patients, adolescent and young adult patients (aged 15–39 years) with brain tumors are likely to have a heterosexual partner at the time of diagnosis. Glioma is more common in adolescent and young adult patients than in pediatric patients and is often treated with temozolomide; before starting radiotherapy or chemotherapy with temozolomide, fertility preservation should be considered (Fig. 9).

#### Clinical Question 1

Which fertility preservation methods can be offered to patients with brain tumors?

Compared with other malignancies, brain tumors more often affect CAYA populations. Surgery-, radiotherapy- or chemotherapy-induced impairment of hypothalamic, pituitary, and gonadal functions can cause sexual dysfunction and/or reduced fertility after treatment. These problems are more serious in younger patients and may only become apparent a long time after complete cure of the underlying malignancy. Therefore, it is important to fully assess the impacts of cancer treatment on reproductive function and to discuss fertility preservation methods before initiation of treatment.

In this CQ, we reviewed optimal fertility preservation methods for patients with brain tumors if i) surgery- and brain irradiation-induced hypothalamic/pituitary dysfunction is expected to cause reproductive dysfunction or ii) chemotherapy or spinal irradiation may directly impair gonadal function.

##### Recommendation 1

Different fertility preservation options are indicated for prepubertal versus postpubertal patients and male versus female patients with brain tumors. Therefore, the recommendation is subdivided according to the individual populations.

1-1. Full pretreatment counselling, posttreatment monitoring of ovarian function, and appropriate estrogen/progestin replacement therapy are recommended for patients who are likely to become infertile only because of hypothalamic/pituitary hypofunction resulting from disease involvement or cancer treatment (e.g., surgery and chemo- and radiotherapy). (Recommendation Grade B).

1-2. Embryo (unfertilized oocyte) cryopreservation is recommended for female patients who have a male partner. (Recommendation Grade B).

1-3. Unfertilized oocyte cryopreservation is recommended for female patients who do not have a male partner. (Recommendation Grade C1).

1-4. Ovarian tissue cryopreservation, although it remains experimental, may be considered in centers with the necessary expertise for female patients who do or do not have a male partner if there is a need for urgent cancer-directed therapy such that there may be limited time for embryo (fertilized oocyte) or unfertilized oocyte cryopreservation or if ovulation induction is difficult for oocyte harvesting (e.g., in prepubertal female patients). (Recommendation Grade C1).

1-5. Use of GnRH agonists for the purpose of fertility preservation is not recommended. (Recommendation Grade C2).

1-6. Sperm cryopreservation is recommended for postpubertal male patients. (Recommendation Grade B).

1-7. No fertility preservation options are currently indicated for prepubertal male patients. (No Recommendation Grade).

#### Clinical Question 2

Is delayed initiation of cancer-director therapy permitted in patients with brain tumors who are interested in fertility preservation to afford them the opportunity to receive a fertility preservation intervention before the start of cancer treatment?

An increasing number of patients with brain tumors achieve remission and consider pregnancy after completing cancer treatment. An increasing number of patients are also interested in fertility preservation before the start of cancer treatment. There are about 150 types of brain tumors, and these tumors are treated in various ways for various durations with treatments that impair fertility to various degrees [2]. Hence, we discussed how long initiation of brain tumor treatment can be delayed if patients request fertility preservation.

##### Recommendation 2

It is desirable to initiate cancer treatment as quickly as possible by taking the optimal timing of treatment of the underlying malignancy and the patient’s status into account. (Recommendation Grade C1).

#### Clinical Question 3

When can a patient with a brain tumor who is interested in having a child consider pregnancy after completing cancer-directed therapy?

With the recent improvement in therapeutic outcomes of brain tumors, an increasing number of young patients with brain tumors are considering pregnancy and are interested in having a child after completing cancer treatment. This question is particularly relevant in patients who are in sustained remission and who have been permitted by their physicians to consider pregnancy. Patients diagnosed with brain tumors, while considering pregnancy may also think about the acceptable timing of pregnancy in relation to cancer treatment. Because patients with brain tumors may have disease- or treatment-related complications, such as hypothalamic/pituitary dysfunction, neurocognitive disorders, and epilepsy, careful counselling is needed for those who are considering pregnancy. Hence, we discussed the acceptable timing for patients with brain tumors to initiate pregnancy attempts.

##### Recommendation 3

3-1. If any teratogenic anti-cancer drug is used, contraception may be considered until the drug and its metabolite(s) are no longer detectable in the body or until the time of corresponding duration has elapsed. (Recommendation Grade C1).

3-2. To determine whether to permit patients with brain tumors to consider pregnancy, a comprehensive evaluation by specialists in related fields may be considered. (Recommendation Grade C1).

## Chapter 8: Digestive system cancers

### General remarks

Digestive system cancers include cancers of the esophagus, stomach, colon, liver, biliary tract, and pancreas. All these cancers frequently affect elderly individuals, and many juvenile-onset cancers of digestive organs are hereditary (Clinical Question 2). If diagnosed at early stages, most digestive system cancers are curable with endoscopic intervention or surgical resection. In patients with advanced disease, health care providers should consider the potential impacts on fertility of intrapelvic surgery or perioperative adjuvant chemo- or chemoradiotherapy (Clinical Questions 1 & 2). In patients with digestive system cancers that are directly invading or metastatic to genital organs (e.g., ovaries) or are unresectable or recurrent, the feasibility of fertility preservation should be carefully assessed by considering the prognosis of the disease (Clinical Questions 1 & 2). In general, only a few of the anticancer drugs frequently used to treat digestive system cancers are highly gonadotoxic, but health care providers should be aware that all such drugs have teratogenicity. Little evidence is available on fertility in patients with digestive system cancers; the fertility preservation methods available for digestive system cancer patients (Clinical Question 3) and the acceptable timing for those patients to consider pregnancy after cancer treatment (Clinical Question 4) are the same as those recommended for patients with cancers of other organs.

#### Clinical Question 1

Which patients with digestive system cancers are eligible for fertility preservation?

Until recently, there have been few active discussions about fertility preservation in patients with digestive system cancers because these cancers more frequently affect elderly individuals and are often refractory to treatment. However, recent improvements in therapeutic outcomes with the advancement of multi-modality therapy and diagnostic procedures has raised public awareness of the importance of fertility preservation in patients with these cancers. However, uncertainties remain about the disadvantages of interventions to preserve fertility in patients with digestive system cancers, and the optimal timing and method for such interventions remain to be established. For patients with digestive system cancers, chemoradiotherapy for rectal cancer has a high risk for infertility in female patients, whereas most standard chemotherapy regimens have an intermediate-to-low risk (ASCO 2013 Clinical Practice Guideline Update). The ASCO Clinical Practice Guideline Update on Fertility Preservation for Patients With Cancer recommends that health care providers caring for patients with cancer should address the possibility of infertility as early as possible before treatment starts. Hence, we extensively reviewed the risk of reduced fertility resulting from treatments for digestive system cancers and the potential disadvantages of fertility preservation methods and thereby focused on colorectal cancer, because it is the cancer where this issue has been addressed most frequently.

##### Recommendation 1

Fertility preservation may be considered in patients scheduled to receive any treatment that is likely to cause infertility, whereby the treatment regimens used and the probability of survival should be taken into account. (Recommendation Grade C1).

#### Clinical Question 2

How should patients with digestive system cancers be counselled regarding fertility preservation?

We performed a literature review to identify the pathological conditions and treatments related to digestive system cancers that require counselling about fertility preservation.

##### Recommendation 2

2-1. Patients diagnosed with hereditary cancer should be counselled about genetic testing and screening for potential synchronous or asynchronous multiple cancers of genital organs and associated fertility problems. (Recommendation Grade B).

2-2. Patients with curable cancers may be educated about the possibility of reduced fertility resulting from operative complications, perioperative adjuvant radiotherapy, and perioperative adjuvant chemotherapy. (Recommendation Grade C1).

2-3. Patients with advanced digestive system cancers that are directly invading or metastatic to genital organs may be counselled regarding the proposed treatments directed toward the genital organs from the aspects of both the expected course and the prognosis of the malignancy and fertility preservation. (Recommendation Grade C1).

#### Clinical Question 3

Which fertility preservation options can be offered to patients with digestive system cancers?

Various fertility preservation options can be offered to CAYA cancer patients, although the level of recommendation varies. Because fertility preservation techniques and options are reviewed in detail in Part 1 of this guideline, in this CQ, we reviewed whether any specific techniques or options can be offered to patients with digestive system cancers.

##### Recommendation 3

In principle, no specific fertility preservation methods are available for patients with digestive system cancers, but this patient population can be offered the same methods as are used in patients with other malignancies (refer to Part 1 of this guideline for further details). The techniques described below may be considered, provided that their indications are carefully assessed and they are used in a safe manner.

3-1. Embryo (unfertilized oocyte) cryopreservation is recommended for female patients who have a male partner. (Recommendation Grade B).

3-2. Unfertilized oocyte cryopreservation may be considered for female patients who do not have a male partner. (Recommendation Grade C1).

3-3. Ovarian tissue cryopreservation, although it remains experimental, may be considered in centers with the necessary expertise for female patients who do or do not have a male partner if there is a need for urgent cancer-directed therapy such that there may be limited time for embryo (fertilized oocyte) or unfertilized oocyte cryopreservation or if ovulation induction is difficult for oocyte harvesting (e.g., in prepubertal females). (Recommendation Grade C1).

3-4. Ovarian transposition (oophoropexy) may be considered in female patients when radiotherapy is performed for rectal cancer. (Recommendation Grade C1).

3-5. Sperm cryopreservation is recommended for male patients. (Recommendation Grade B).

3-6. Nerve-sparing surgery is recommended if surgery is likely to cause erectile/ejaculatory dysfunction. (Recommendation Grade B).

#### Clinical Question 4

When can a patient with digestive system cancer who is interested in having a child consider pregnancy after completing cancer-directed therapy?

Multi-modality therapy, which primarily comprises surgery, chemotherapy, and radiotherapy, is used to treat digestive system cancers, and its impacts on subsequent conception and pregnancy remain to be determined. For patients interested in having a child after completing cancer-directed therapy, the question when they are permitted to consider pregnancy after completion of cancer treatment is particularly important.

##### Recommendation 4

If any anti-cancer drug used is teratogenic, contraception may be considered until the drug and its metabolite(s) are no longer detectable in the body or until they can assume to no longer be present on the basis of the drug half-life. (Recommendation Grade C1).

Guideline Review Committee

## Supplementary Information

Below is the link to the electronic supplementary material.

Supplementary file1 (DS_Store 6 kb)
