# Correction to: Japan Society of Clinical Oncology Clinical Practice Guidelines 2017 for fertility preservation in childhood, adolescent, and young adult cancer patients: part 2

**DOI:** 10.1007/s10147-022-02137-5

**Published:** 2022-02-13

**Authors:** Akiko Tozawa, Fuminori Kimura, Yasushi Takai, Takeshi Nakajima, Kimio Ushijima, Hiroaki Kobayashi, Toyomi Satoh, Miyuki Harada, Kohei Sugimoto, Shigehira Saji, Chikako Shimizu, Kyoko Akiyama, Hiroko Bando, Akira Kuwahara, Tatsuro Furui, Hiroshi Okada, Koji Kawai, Nobuo Shinohara, Koichi Nagao, Michio Kitajima, Souichi Suenobu, Toshinori Soejima, Mitsuru Miyachi, Yoko Miyoshi, Akihiro Yoneda, Akihito Horie, Yasushi Ishida, Noriko Usui, Yoshinobu Kanda, Nobuharu Fujii, Makoto Endo, Robert Nakayama, Manabu Hoshi, Tsukasa Yonemoto, Chikako Kiyotani, Natsuko Okita, Eishi Baba, Manabu Muto, Iwaho Kikuchi, Ken-ichirou Morishige, Koichiro Tsugawa, Hiroyuki Nishiyama, Hajime Hosoi, Mitsune Tanimoto, Akira Kawai, Kazuhiko Sugiyama, Narikazu Boku, Masato Yonemura, Naoko Hayashi, Daisuke Aoki, Nao Suzuki, Yutaka Osuga

**Affiliations:** 1 Department of Obstetrics and Gynecology, St. Marianna University School of Medicine, 2-16-1 Sugao, Miyamae-ku, Kawasaki-shi, Kawasaki, Kanagawa 216-8511 Japan; 2 Department of Obstetrics and Gynecology, Shiga University of Medical Science, Seta Tsukinowa-Cho Otsu, Shiga, 520-2192 Japan; 3 Department of Obstetrics and Gynecology Saitama Medical Center, Saitama Medical University, 1981 Kamoda, Kawagoe City, Saitama 350-3550 Japan; 4 Department of Endoscopy, Gastrointestinal Endoscopy Division, National Cancer Center Hospital, 5-1-1 Tsukiji, Chuo-ku, Tokyo, 104-0045 Japan; 5 Department of Obstetrics and Gynecology, Kurume University School of Medicine, 67 Asahi-machi, Kurume, Fukuoka, 830-0011 Japan; 6 Department of Obstetrics and Gynecology, Graduate School of Medical and Dental Sciences, Kagoshima University, 8-35-1 Sakuragaoka, Kagoshima, 890-8520 Japan; 7 Department of Obstetrics and Gynecology, Faculty of Medicine, University of Tsukuba, Tennoudai, Tsukuba, Ibaraki 305-8575 Japan; 8 Department of Obstetrics and Gynecology, Graduate School of Medicine, The University of Tokyo, 7-3-1, Hongo, Bunkyo, Tokyo, 113-8655 Japan; 9 International Center for Reproductive Medicine, Dokkyo Medical University Saitama Medical Center, 2-1-50 Minamikoshigaya, Koshigaya, Saitama 343-8555 Japan; 10 Department of Medical Oncology, Fukushima Medical University, 1 Hikarigaoka, Fukushima City, Fukushima 960-1295 Japan; 11 Department of Breast and Medical Oncology, National Center for Global Health and Medicine, 1-21-1 Toyama, Shinjuku-ku, Tokyo, 162-8655 Japan; 12 Department of Breast and Endocrine Surgery, St. Marianna University School of Medicine, 2-16-1 Sugao, Miyamae, Kawasaki, Kanagawa 216-8511 Japan; 13 Department of Breast and Endocrine Surgery, Faculty of Medicine, University of Tsukuba, Tennoudai, Tsukuba, Ibaraki 305-8575 Japan; 14 Department of Obstetrics and Gynecology, Graduate School of Biomedical Sciences, Tokushima University, 3-18-15, Kuramoto-cho, Tokushima, 770-8503 Japan; 15 Department of Obstetrics and Gynecology, Gifu University Graduate School of Medicine, 1-1, Yanagido, Gifu City, Gifu 501-1194 Japan; 16 Department of Urology, International University of Health and Welfare, 852, Hatakeda Narita, Chiba, 286-0124 Japan; 17 Department of Renal and Genitourinary Surgery, Hokkaido University Graduate School of Medicine, Kita 15Nishi 7, Kita-ku, Sapporo, Hokkaido 060-8638 Japan; 18 Department of Urology, Toho University Faculty of Medicine, 6-11-1, Omori-Nishi, Ota-ku, Tokyo, 143-8541 Japan; 19 Department of Obstetrics and Gynecology, Nagasaki University Graduate School of Biomedical Sciences, Nagasaki, Japan; 20 Division of General Pediatrics and Emergency Medicine, Department of Pediatrics, Oita University Faculty of Medicine, 1-1 Idaigaoka, Hasama, Yufu, Oita, 879-5593 Japan; 21 Department of Radiation Oncology, Kobe Proton Center, 1-6-8, Minatojima-minamimachi, Chuo-ku, Kobe City, Hyogo 650-0047 Japan; 22 Department of Pediatrics, Kyoto Prefectural University of Medicine Graduate School of Medical Science, 465 Kajii-cho, Hirokoji, Kamigyo-ku, Kyoto, 602-8566 Japan; 23 Department of Health and Nutrition, Faculty of Health and Nutrition, Osaka Shoin Women’s University, 4-2-26 Hishiya-nishi, Higashi-Osaka, Osaka, 577-8550 Japan; 24 Division of Surgery/Surgical Oncology, National Center for Child Health and Development, Tokyo, Japan; 25 Division of Pediatric Surgical Oncology, National Cancer Center Hospital, 5-1-1 Tsukiji, Chuo-ku, Tokyo, 104-0045 Japan; 26 Department of Gynecology and Obstetrics, Kyoto University Graduate School of Medicine, 54 Kawahara-cho, Shogoin, Sakyoku, Kyoto, 606-8507 Japan; 27 Pediatric Medical Center, Ehime Prefectural Central Hospital, 83 Kasuga-machi, Matsuyama City, Ehime 790-0024 Japan; 28 Division of Clinical Oncology and Hematology, Department of Internal Medicine, The Jikei University School of Medicine, 3-19-18 Nishi-Shinbashi, Minato-ku, Tokyo, 105-8461 Japan; 29 Division of Hematology, Department of Medicine, Jichi Medical University, 1-847 Amanuma, Omiya-ku, Saitama City, Saitama 330-8503 Japan; 30 Division of Transfusion, Okayama University Hospital, 2-5-1 Shikata-cho, Kita-ku, Okayama, 700-8558 Japan; 31 Department of Orthopaedic Surgery, Kyushu University, Maidashi 3-1-1, Higashi-ku, Fukuoka, 812-8582 Japan; 32 Department of Orthopaedic Surgery, Keio University School of Medicine, 35 Shinanomachi, Shinjuku, Tokyo, 160-8582 Japan; 33 Department of Orthopedic Surgery, Osaka City University Graduate School of Medicine, 1-4-3 Asahi-Machi, Abeno-Ku, Osaka, 545-8585 Japan; 34 Division of Orthopedic Surgery, Chiba Cancer Center, 666-2 Nitona-cho, Chuo-ku, Chiba, 260-8717 Japan; 35 Children’s Cancer Center, National Center for Child Health and Development, 2-10-1 Okura, Setagaya-ku, Tokyo, 157-8535 Japan; 36 Department of Gastrointestinal Medical Oncology, National Cancer Center Hospital, 5-1-1 Tsukiji, Chuo-ku, Tokyo, 104-0045 Japan; 37 Department of Oncology and Social Medicine, Graduate School of Medical Sciences, Kyushu University, 3-1-1 Maidashi, Higashi-ku, Fukuoka, 812-8582 Japan; 38 Department of Therapeutic Oncology, Kyoto University Graduate School of Medicine, 54 Kawahara-cho, Shogoin, Sakyoku, Kyoto, 606-8507 Japan; 39 Department of Obstetrics and Gynecology, Medical Park Yokohama, 1-1-8, Sakuragi-cho, Yokohama, Kanagawa 231-0062 Japan; 40 Department of Urology, Faculty of Medicine, University of Tsukuba, Tennoudai, Tsukuba, Ibaraki 305-8575 Japan; 41 Department of Nursing, Doshisha Women’s College of Liberal Arts, Kodo, Kyotanabe City, Kyoto 610-0395 Japan; 42 Chugoku Central Hospital, 148-13, Kamiiwanari, Miyuki-cho, Fukuyama City, Hiroshima 720-0001 Japan; 43 Department of Musculoskeletal Oncology and Rehabilitation Medicine, National Cancer Center Hospital, 5-1-1 Tsukiji, Chuo-ku, Tokyo, 104-0045 Japan; 44 Department of Clinical Oncology and Neuro-Oncology Program, Hiroshima University Hospital, 1-2-3 Kasumi, Minami-ku, Hiroshima, 734-8551 Japan; 45 Department of Medical Oncology and General Medicine, IMSUT Hospital, Institute of Medical Science, University of Tokyo, 4-6-1, Shirokanedai, Minato-ku, Tokyo, 108-8639 Japan; 46 Department of Pharmacy, National Cancer Center Hospital East, 6-5-1 Kashiwanoha, Kashiwa-shi, Chiba, Japan; 47 Graduate School of Nursing Science, St Luke’s International University, 10-1 Akashi-cho, Chuo-ku, Tokyo, 104-0044 Japan; 48 Department of Obstetrics and Gynecology, Keio University School of Medicine, 35 Shinanomachi, Shinjuku, Tokyo, 160-8582 Japan

## Correction to: International Journal of Clinical Oncology 10.1007/s10147-021-02076-7

The article, Japan Society of Clinical Oncology Clinical Practice Guidelines 2017 for fertility preservation in childhood, adolescent, and young adult cancer patients: part 2 written by Akiko Tozawa, Fuminori Kimura, Yasushi Takai, Takeshi Nakajima, Kimio Ushijima, Hiroaki Kobayashi, Toyomi Satoh, Miyuki Harada, Kohei Sugimoto, Shigehira Saji, Chikako Shimizu, Kyoko Akiyama, Hiroko Bando, Akira Kuwahara, Tatsuro Furui, Hiroshi Okada, Koji Kawai, Nobuo Shinohara, Koichi Nagao, Michio Kitajima, Souichi Suenobu,Toshinori Soejima, Mitsuru Miyachi, Yoko Miyoshi, Akihiro Yoneda, Akihito Horie,Yasushi Ishida, Noriko Usui, Yoshinobu Kanda, Nobuharu Fujii, Makoto Endo, Robert Nakayama, Manabu Hoshi, Tsukasa Yonemoto, Chikako Kiyotani, Natsuko Okita, Eishi Baba, Manabu Muto, Iwaho Kikuchi, Ken‑ichirou Morishige, Koichiro Tsugawa, Hiroyuki Nishiyama, Hajime Hosoi, Mitsune Tanimoto, Akira Kawai, Kazuhiko Sugiyama, Narikazu Boku, Masato Yonemura, Naoko Hayashi, Daisuke Aoki, Nao Suzuki, Yutaka Osuga was originally published Online First without Open Access.

With the author(s)’ decision to opt for Open Choice the copyright of the article changed on February 14, 2022 to © Author(s) 2021 and the article is forthwith distributed under a Creative Commons Attribution 4.0 International License, which permits use, sharing, adaptation, distribution and reproduction in any medium or format, as long as you give appropriate credit to the original author(s) and the source, provide a link to the Creative Commons licence, and indicate if changes were made. The images or other third party material in this article are included in the article’s Creative Commons licence, unless indicated otherwise in a credit line to the material. If material is not included in the article’s Creative Commons licence and your intended use is not permitted by statutory regulation or exceeds the permitted use, you will need to obtain permission directly from the copyright holder. To view a copy of this licence, visit http://creativecommons.org/licenses/by/4.0.

The original article has been corrected.

